# Assessing soil CO_2_ emission on eucalyptus species using UAV-based reflectance and vegetation indices

**DOI:** 10.1038/s41598-024-71430-2

**Published:** 2024-08-31

**Authors:** Fernando Saragosa Rossi, João Lucas Della-Silva, Larissa Pereira Ribeiro Teodoro, Paulo Eduardo Teodoro, Dthenifer Cordeiro Santana, Fábio Henrique Rojo Baio, Wendel Bueno Morinigo, Luís Guilherme Teixeira Crusiol, Newton La Scala, Carlos Antonio da Silva

**Affiliations:** 1 PPG-Ciência do Solo, State University of São Paulo (UNESP), Jaboticabal, 15385-000 Brazil; 2 PPG-Bionorte, State University of Mato Grosso (UNEMAT), Sinop, 78550-000 Brazil; 3 Federal University of Mato Grosso do Sul (UFMS), Chapadão do Sul, 79560-000 Brazil; 4 PPGCAM, Federal University of Mato Grosso (UFMT), Sinop, 78555000 Brazil; 5 National Soybean Research Center (Embrapa Soja), Brazilian Agricultural Research Corporation, Londrina, 86001–970 Brazil; 6 Department of Geography, State University of Mato Grosso (UNEMAT), Sinop, Mato Grosso 78555000 Brazil

**Keywords:** Plant sciences, Environmental sciences

## Abstract

Eucalyptus species play an important role in the global carbon cycle, especially in reducing the greenhouse effect as well as storing atmospheric CO₂. Thus, assessing the amount of CO₂ released by the soil in forest areas can generate important information for environmental monitoring. This study aims to verify the relation between soil carbon dioxide (CO₂) flux (FCO₂), spectral bands, and vegetation indices (VIs) derived from a UAV-based multispectral camera over an area of eucalyptus species. Multispectral imageries (green, red-edge, and near-infrared) from the Parrot Sequoia sensor, derived vegetation indices, and the FCO₂ data from a LI-COR 8100 analyzer, combined with soil moisture and temperature data, were collected and related. The vegetation indices ATSAVI (Adjusted Transformed Soil-Adjusted VI), GSAVI (Green Soil Adjusted Vegetation Index), and SAVI (Soil-Adjusted Vegetation Index), which use soil correction factors, exhibited a strong negative correlation with FCO₂ for the species *E. camaldulensis*, *E. saligna*, and *E. urophylla* species. A Multivariate Analysis of Variance showed significance (*p* < 0.01) for the species factor, which indicates that there are differences when considering all variables simultaneously. The results achieved in this study show a specific correlation between the data of soil CO₂ emission and the eucalypt species, providing a distinction of values between the species in the statistical data.

## Introduction

The increasing demand for timber over the last few years has led to land use change to areas dedicated to tree crops with rapid growth^1^. In Brazil, the total area dedicated to planted forest is about 9.5 million hectares (ha), 76.9% of which are eucalyptus (Eucalyptus spp.) cash crops^2^. This genus is distinguished by the availability of cultivars adapted to the different Brazilian biomes and edaphoclimatic conditions^3^. Eucalyptus monoculture fields are distributed throughout the tropics and subtropics, notably in Brazil^4^. Mato Grosso do Sul state has been the eucalyptus expansion head, presenting a significant increase in cellulose production in the last five years, making this state the leading exporter in the first four months of 2020^5^.

Eucalyptus has a greater potential to stock atmospheric carbon in aerial biomass or soil, mainly when associated with pasture or annual crops and especially in the conversion of degraded land to productive land and for renewable energy sources^6^. Thus, forest crops play an important role in the global carbon cycle, especially in reducing the greenhouse effect as well as stocking atmospheric CO₂^7^. Monitoring the amount of CO₂ released by the soil in forest areas can generate important information for cultivation, being influenced by the type of management and the effects of human actions on carbon emission, as well as climatic variation, since soil respiration is the second largest flux of carbon between terrestrial ecosystems and the atmosphere^8^. Soil CO₂ emission (FCO₂) in eucalyptus crops is affected by environmental factors, such as soil temperature, water content, and precipitation variations^9^.

Throughout the carbon cycle, CO₂ flow is based on plant (autotrophic) and microbial respiration (heterotrophic). Soil respiration comprises the autotrophic respiration below the soil surface where plant roots are the main contributors, and the heterotrophic respiration of microorganisms during the decomposition of organic matter^10–12^. The challenges of forest management rely on reliable, accurate, and cost-effective methods to adequately report forest dynamics, especially because of the growing carbon trading (e.g., reducing emissions from deforestation and forest degradation)^13^. In this sense, remote sensing techniques may assist in understanding carbon dynamics comprehension and the role of greenhouse gases (GHGs) in the soil-atmosphere interface, for instance through multispectral image analysis in conjunction with in situ measurements for the monitoring, mapping, and enforcement of vegetation cover^14,15^.

Significant changes have occurred in forestry activities management, considering the advancement of big data, geoprocessing, and informatics technologies^16^, as well as the use of remotely piloted drones that use images for crop mapping, evaluation of cultivated areas, disease detection, and soil mapping^17^. Thus, image analysis tools through spectral indices are presented as alternatives for further research and decision-making more objectively. There is a relationship between the amount of cumulative FCO₂ and the growth of eucalyptus trees since these plants need the nutrients released into the soil after decomposition of the existing organic material^18^. From the perspective of remote sensing techniques, vegetation indices are capable of diagnosing certain factors related to eucalyptus plants, such as the amount of water in the leaf^19^, leaf area index^20^, and distinguishing species by reflectance and growth^16^. Thus, this manuscript hypothesizes that differences in FCO₂ may indicate the existence of a correlation with the spectral behavior of eucalyptus species and that the UAV-based image sensor may be a proxy for assessing CO₂ flux. The principal contributions of this paper can be summarized as follows:

1. Evaluation of the relationship between soil carbon dioxide flux (FCO₂) and UAV-based multispectral data.
2. Assessment of the correlation between reflectance values and derived vegetation indices with the spectral behavior of eucalyptus species.
3. Investigation of whether differences in FCO₂ indicate a correlation with the spectral behavior of eucalyptus species.
4. Testing of the hypothesis that UAV-based image sensors can serve as proxies for assessing CO₂ flux in eucalyptus species.

## Results

Table 1 presents the descriptive statistics for the variables FCO₂, M_s_, and T_s_, concerning the eucalyptus species. The mean value obtained for FCO₂ was 0.54 µmol m^−2^ s^−1^, with minimum and maximum values ranging from 0.1 to 1.65 µmol m^−2^ s^−1^. For M_s_ and T_s_, the mean values were 8.28 and 20.9, respectively. The coefficient of variation was below 10% for T_s_ and less than 30% for M_s_. However, for FCO₂, this value was 56%. Therefore, only temperature ex-hibited low variability (CV < 10%), remaining consistent with the minimum and maximum val-ues, while the other variables displayed moderate to high variability in their values.

**Table 1:** Descriptive statistics of soil emissions (FCO₂), soil moisture (M_s_), and soil temperature (T_s_) in relation to six eucalyptus species in the year 2021.

	FCO₂	M_s_	T_s_
Mean	0.54	8.28	20.9
Median	0.46	8.0	21.0
Maximum	1.65	15.0	24.0
Minimum	0.1	3.0	18.0
Range	1.55	12.0	6.2
Standard deviation	0.3	2.4	1.7
Coefficient of variation (%)	56	29	8

**Fig. 1:**
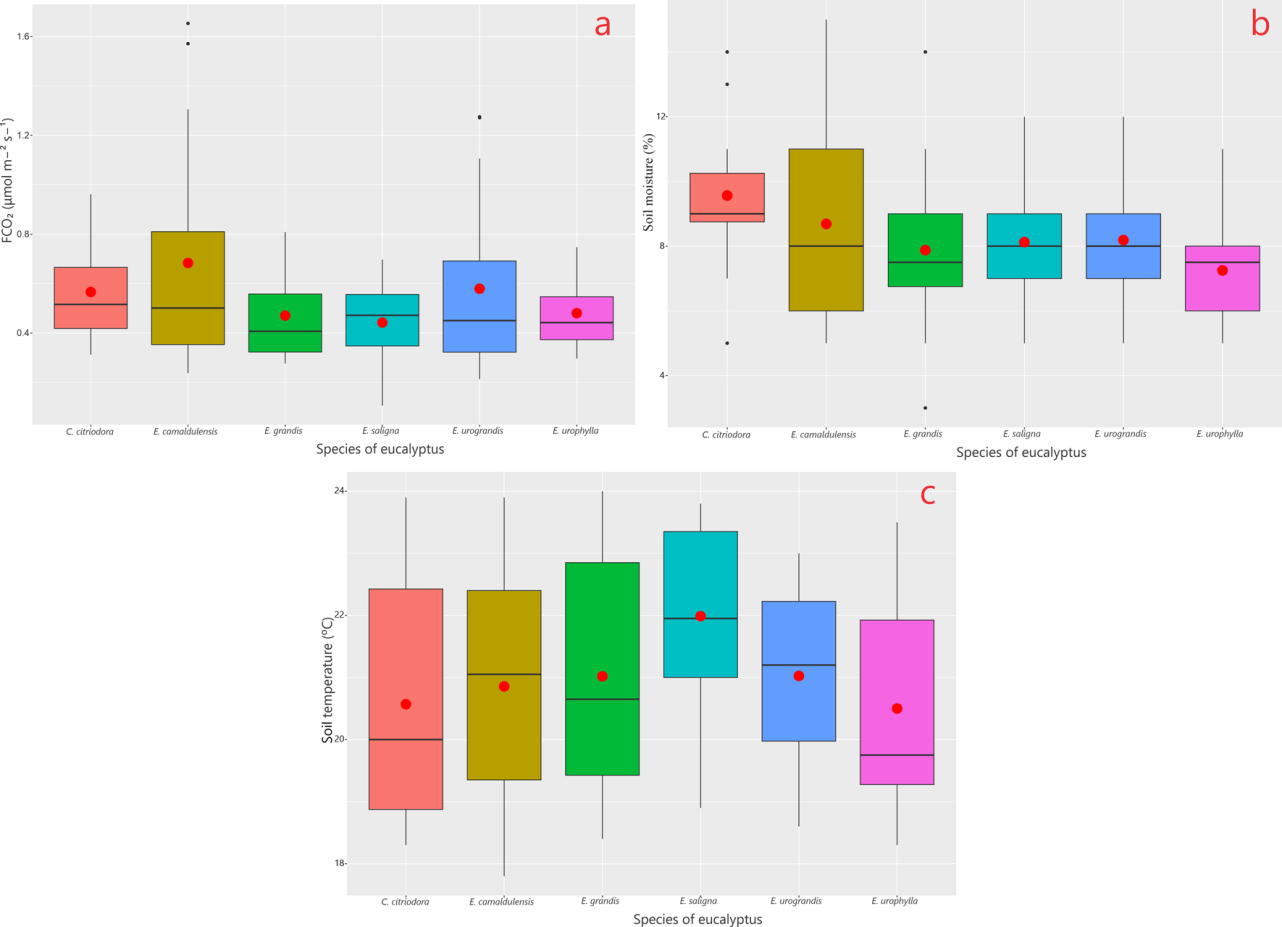
Boxplot of soil CO₂ emissions (FCO₂), soil moisture (M_s_) and soil temperature (T_s_) values concerning the eucalyptus species.

Figure 1 depicts the boxplot for the statistical analysis of data distribution for the six eucalyptus species concerning the variables FCO₂, M_s_, and T_s_. The asymmetric position of the median relative to the mean symbolizes an association with non-parametric data, which are closer to both the lower quartile (Q1) and upper quartile (Q3) in both representations. The eucalyptus species exhibit different ranges of values for the measurements and the presence of outliers for the species *E. camaldulensis* and *E. urograndis* in the case of the FCO₂ variable (Fig. 1a), and *Corymbia citriodora* and *E. grandis* for M_s_, represented by the points that deviate from the box edges.

Among the species evaluated for the FCO₂ variable, *E. saligna* and *E. urophylla* stand out for having values distributed more closely around the mean, meaning there is a proximity be-tween the median and mean values. This occurrence is also observed for the M_s_ variable (Fig. 1b) and T_s_ variable (Fig. 1c) for the species *E. saligna*, *E. urograndis*, *E. urophylla*, and *E. saligna*, respectively.

For the species *E. camaldulensis* and *E. urograndis* (FCO₂), and *C. citriodora* and *E. grandis* (M_s_), the median is below the mean, possibly due to the outliers that negatively impacted the mean. In general, the interquartile ranges, representing the interval between the second and third quartiles, are larger for all variables than the interquartile range between the first and second quartiles, indicating greater data dispersion among the top 25% of observed values.

Pearson's correlation analysis (Figure S1) was high and positive between the features derived from the aggregated photosynthetic processes. The green, red, red-edge, and NIR (near infrared) spectral bands had a negative linear correlation with FCO₂. The GEMI (Global Environment Monitoring Index), ATSAVI (Adjusted Transformed soil-adjusted VI), SAVI (Soil-Adjusted Vegetation Index), EVI2 (Enhanced Vegetation Index 2), GSAVI (Green Soil Adjusted Vegetation Index), GDVI (Difference NIR/Green Difference Vegetation Index), CIrededge (Chlorophyll Index RedEdge), CIgreen (Chlorophyll Index Green) and BWDRVI (Blue-wide dynamic range vegetation index) indices showed the highest correlation with the characteristics FCO₂. The FCO₂ variable showed links with all variables, but no variable was clearly associated.

Considering the large number of variables acquired via remote sensing and assigned in situ to the eucalyptus species applied in Pearson’s correlation analysis, the variables GEMI, ATSAVI, SAVI, EVI2, GSAVI, GDVI, CIrededge, CIgreen and BWDRVI were selected, which had the highest correlations with FCO₂.

Multivariate analysis of variance for eucalyptus species, the variables FCO₂, M_s_, T_s_, the spectral bands (green, red, NIR and red edge) and vegetation indices (GEMI, ATSAVI, SAVI, EVI2, GSAVI, GDVI, CIrededge, CIgreen and BWDRVI) were used to test the hypothesis of the difference between the eucalypt species. The significance level for the species considering all variables simultaneously was ρ =  < 0.001 for Pillai's test, showing that the null hypothesis is rejected, as there are differences among eucalyptus species.

**Fig. 2:**
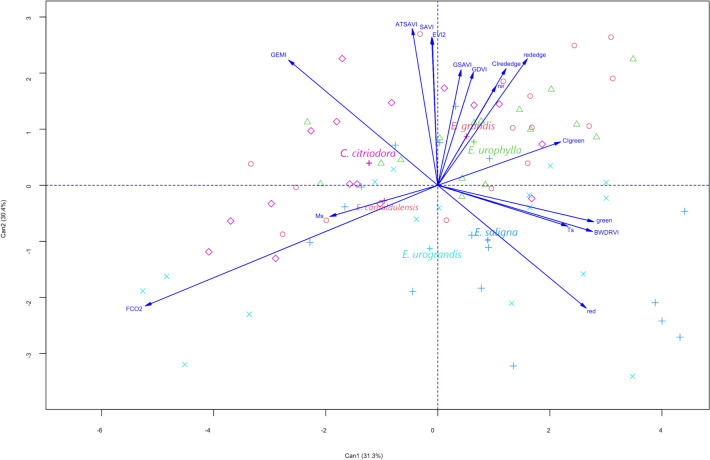
Two-dimension plot of Can1 versus Can2 scores generated by the canonical variable analysis considering eucalyptus species and FCO_2_, soil moisture (Ms) and temperature (Ts), spectral bands (green, red, NIR, and red-edge), and vegetation indices (GEMI, ATSAVI, SAVI, EVI2, GSAVI, GDVI, CIrededge, CIgreen and BWDRVI).

The canonical analysis considers the eucalyptus species and the variables with a strong correlation to FCO₂ (Fig. 2). The ratio of the variance accumulated in the first two canonical variables was higher than 61.7% (Can1 and Can2). The variables FCO₂, M_s_, GSAVI, GDVI, CIrededge, NIR, rededge and CIgreen were most important in distinguishing eucalyptus species in Can1, while part red, green, BWDRVI, T_s_, GEMI, ATSAVI, SAVI and EVI2 ratio were the most relevant variables in Can2. This finding reveals that a significant portion of the variability of the eucalypt species concerning the variables evaluated in each species can be summarized in two linear combinations, named Can1 and Can2.

From the analyzed variables in Fig. 2, it can be observed that red, rededge, ATSAVI, GEMI and FCO₂ presented the greatest variability, which can be proven by the size of the module of their vectors. There are high correlations between the variables FCO₂ and M_s_; red edge, NIR, CIrededge, GSAVI, GDVI, ATSAVI, SAVI and EVI2; T_s_, green and BWDRVI for forming acute angles between the variables. There is no other correlation combination between the variables, as it forms an angle close to 90 degrees, as shown in Fig. 2. The set of variables FCO₂ and M_s_; CIrededge and red-edge; red; GEMI were those that contributed most to the discrimination of the species *E. camaldulensis*; *E. grandis* and *E. urophylla*, *E. saligna* and *C. citriodora*, respectively. On the other hand, the species *E. urophylla* and *E. grandis* were more similar in the canonical variables analysis.

**Fig. 3:**
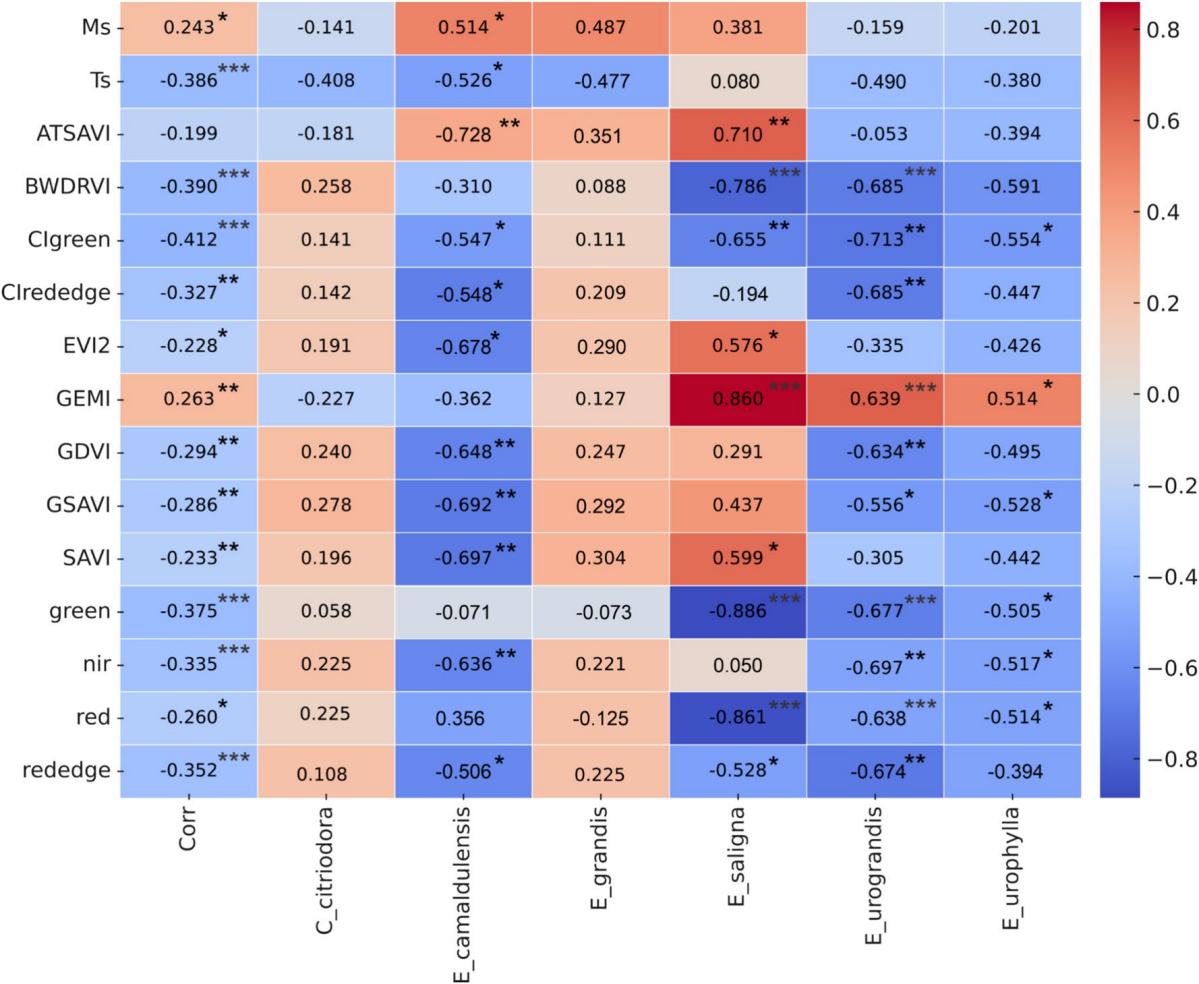
Heatmap of correlations between eucalyptus species and FCO_2_ variables as per the modeling of canonical variable analysis.

In Fig. 3, we exclusively present the zoomed-in view of the direct correlation between the variables and FCO₂, which encompasses the correlations established among the species of eucalyptus species showed specific correlations with FCO₂. Statistical significance is considered at *p* ≤ 0.05 by *, *p* ≤ 0.01 by ** and *p* ≤ 0.001 by ***. The FCO₂ correlation to M_s_ (0.243*), EVI2 (− 0.228*), red (− 0.260*), SAVI (− 0.233*), CIrededge (− 0.327**), GEMI (0.263**), GDVI (− 0.294**), GSAVI (− 0.286**), T_s_ (− 0.386***), BWDRVI (− 0.390***), CIgreen (− 0.412***), green (− 0.375***), NIR (− 0.335***) and rededge (− 0.352***), with specific correlation among *E. camaldulensis* (0.514*) and *E. grandis* (0.487*); *E. camaldulensis* (− 0.678**) and *E. saligna* (0.576*); *E. saligna* (− 0.861***), *E. urograndis* (− 0.638**) and *E. urophylla* (− 0.514*); *E. camaldulensis* (− 0.697**) and *E. saligna* (0.599*); *E. camaldulensis* (− 0.548*) and *E. urograndis* (− 0.685**); *E. saligna* (0.860***), *E. urograndis* (0.639**) and *E. urophylla* (0.514*); *E. camaldulensis* (− 0.648**) and *E. urograndis* (− 0.634**); *E. camaldulensis* (− 0.692**), *E. urograndis* (− 0.556*) and *E. urophylla* (− 0.528*); *E. camaldulensis* (− 0.526*); *E. saligna* (− 0.786***), *E. urograndis* (− 0.685**) and *E. urophylla* (− 0.591*); *E. camaldulensis* (− 0.547*), *E. saligna* (− 0.655**), *E. urograndis* (− 0.713**) and *E. urophylla* (− 0.554*); *E. saligna* (− 0.886***), *E. urograndis* (− 0.677**) and *E. urophylla* (− 0.505*); E. camaldulensis (− 0.636**), *E. urograndis* (− 0.697**) and *E. urophylla* (− 0.517*); *E. camaldulensis* (− 0.506*), *E. saligna* (− 0.528*) and *E. urograndis* (− 0.674**) respectively were significant at 99%. For the variable ATSAVI, there was no correlation with FCO₂, however, it displayed specific correlations with E. camaldulensis (− 0.728**) and *E. saligna* (0.710**).

*C. citriodora* had no significance for the correlation to FCO₂ in the entire set of variables. The species most often correlated significantly with FCO₂ were *E. camaldulensis* and *E. saligna*.

## Discussion

An absence of strong and significant correlations between FCO₂ and the rest of the variables in a general set with eucalyptus species is noticeable (Figure S1). However, differences were identified among the eucalyptus species while simultaneously considering the soil variables with the main vegetation indices.

As there is a low and negative correlation between the variables and FCO_2_, we can suggest that the variables move in opposite directions, which means that when the values of a variable increase, FCO_2_ tends to decrease, and therefore other factors may be influencing this relationship, since leaves are the most important organs for the spectral characterization of vegetation, reflecting the conditions of the plant^21^.

The interactions of a species during its cycle with the environment are known to be complex, with many aspects not being sufficiently clarified^22^. This may explain the behavior of the *C. citriodora* species response when correlated with the FCO_2_ variable, which is different from what was found for the other eucalyptus species. These measurements and comparisons provide a better understanding of plant responses to multispectral variables. As a fast-growing species, eucalyptus plays an important role in mitigating climate change due to its ability to absorb carbon dioxide^23^.

Scientific research based on field data and remote sensing, as evidenced in previous studies, Teodoro et al.^24^, Oliveira et al.^16^, and Xu et al.^25^ are important tools for future planning and decision-making. These tools are fundamental in reducing climate risks that directly affect biodiversity, agriculture, water resources, coastal zones, etc., as well as promoting sustainable development by allowing forestry practices to be carried out more safely.

Total soil respiration comprises autotrophic and heterotrophic respiration^26,27^, which are directly and indirectly related to biotic and abiotic factors^28,29^ for the regulation of an ecosystem. The behavior of autotrophic respiration is strongly correlated with photosynthesis^30,31^ and is mediated by phenological dynamics throughout the plant cycle^32,33^. Heterotrophic respiration from the mineralization of organic matter by the decomposer community is highly affected by soil carbon availability, moisture and temperature^34,35^.

M_s_ and T_s_ are important for the respiration process of soils and directly influence the decomposition of organic matter and microbial and root activities^36,37^. Thus, M_s_ showed a positive correlation and T_s_ showed a negative correlation with FCO₂. However, this low-magnitude correlation between M_s_ and T_s_ with FCO₂ can be explained by the low variation over the time series period^38^. The effects of T_s_ on FCO₂ may be partially hidden by the effect of M_s_, since these are interdependent variables and commonly change simultaneously^39^.

The results obtained by Almeida et al.^40^, Dossou-Yovo et al.^41^ and Pires et al.^42^ show that the emission process and the transport of the gas from the soil interior to the atmosphere depend on the porosity, the moisture content and the conditions of the whole soil biota. These conditions in the ecosystem are at the same time very favorable for the activity of microorganisms, which can positively influence respiration rates^43^. In addition, management practices will influence the behavior of carbon storage capacity and the loss of CO₂ from the soil to the atmosphere^44^.

In the case of data obtained via passive remote sensors, the spectral bands green, NIR, red, and red-edge were negatively correlated and of low magnitude with FCO₂, but with significance in *E. camaldulensis*, *E. saligna*, and *C. citriodora* (Fig. 3). The low association between spectral bands and FCO₂ is directly associated with the green vegetative canopy and also related to the stage of development of the eucalyptus species that influences leaf thickness, water content and response of leaf chlorophyll contents. The chlorophyll a and b pigmentations are the ones that most influence the electromagnetic radiation in the visible region, with two absorption peaks, the higher in the red band and the lower in the blue band, respectively^45,46^, favoring a better result for the classifier that used the red (R) and blue (B) spectral bands. From Silva et al.^47^, the green wavelength response is entirely associated with the potential of photosynthetic activity, and this relation is directly related to the utilization of available radiation by these chlorophyll pigments. The process of CO₂ absorption from the atmosphere by the leaves occurs when the pores of the stomas open, allowing the carbon dioxide to enter the leaf, mainly located in the lower epidermis^48,49^.

The use of the red and near-infrared (NIR) spectral bands in the composition of vegetation indices is because these wavelengths are inversely proportional to leaf reflectance, with greater than 90% sensitivity on the spectral behavior of a vegetation canopy^50^. BWDRVI and GEMI uses blue and NIR spectral reflectance to measure the density and greenness of vegetation, showing a specific negative and positive correlation with *E. saligna*, *E. urograndis* and *E. uroplylla*, respectively. The blue spectral band is poorly absorbed by the upper part of the plant canopy for the process of photosynthesis, the lower levels of the canopy do not contribute to reflectance and scattering of electromagnetic radiation, which impairs the correlation of BWDRVI and GEMI with FCO₂.

The absorption of the red radiation is by the photosynthesizing pigments of the leaves, which are the chlorophyll ‘*a*’, while in the near-infrared the absorption is almost zero, and the reflectance and transmittance are high, due to the internal cellular structures of the leaves and the effect of the multiple reflectances^51^. In previous studies, the ratio between leaf chlorophyll and carotenoid can be considered as a proxy to track the physiological and phenological status of vegetation^52^, but its non-obvious absorption characteristics in the visible spectral regions^53^. To capture variations in the amount of chlorophyll not only in the upper canopy but also throughout the foliage, the EVI2, ATSAVI and SAVI index were calculated using a combination of the NIR and red-edge bands. A negative, positive, and specific negative correlation with *E. camaldulensis* and *E. saligna*, respectively, differed from the correlations of the EVI2, ATSAVI and SAVI index, which can be explained by the low values of chlorophyll content.

SAVI, GSAVI and ATSAVI use a soil brightness correction factor (L) applied to maximize the reduction of soil effects on the vegetation signal and showed negative and positive correlations with FCO₂ for *E. camaldulensis* and *E. saligna*, respectively. The constant L value was 0.5, recommended for a wide range of vegetation scenarios^54^, but using the value of L = 0.5 in other studies resulted in a higher standard deviation in pixel values^55^. SAVI, GSAVI and ATSAVI put down general vegetation characteristics by correcting for ground brightness, and is a disadvantage when vegetation is at peak vegetation^56^. However, the presence of intact dead organic matter on the soil surface decreased the effect of precipitation on FCO₂, as it has no effect on the soil structure and only during its decomposition, thus being a physical barrier against the abrupt entry of water into the soil^57^. The presence of decomposing dead plant matter forms aggregating and stabilizing substances in the soil structure, with beneficial effects on increasing soil carbon stocks through physical, chemical, and biological processes^58,59^. However, there is evidence that dead organic matter increases soil CO₂ emissions since straw can be rapidly broken down by soil microorganisms^60,61^. More research is needed, in order to validate the effects of straw on soil CO₂ emissions.

These results enable the management of differentiation among eucalyptus species using sensors coupled to remotely piloted drone and FCO₂, thus integrating new assumptions in this line of research. The reflectance of eucalyptus species acquired by remotely piloted aircraft makes it possible to obtain data about them in a fast, non-destructive, and accurate way. Measuring CO₂ provides important information about the carbon emissions in areas of eucalyptus. Both pieces of information contribute to the improvement of forest management. It is important to stress that new studies should be conducted in places with different soil and climate conditions from the current research.

## Methods

### Studied area

**Fig. 4:**
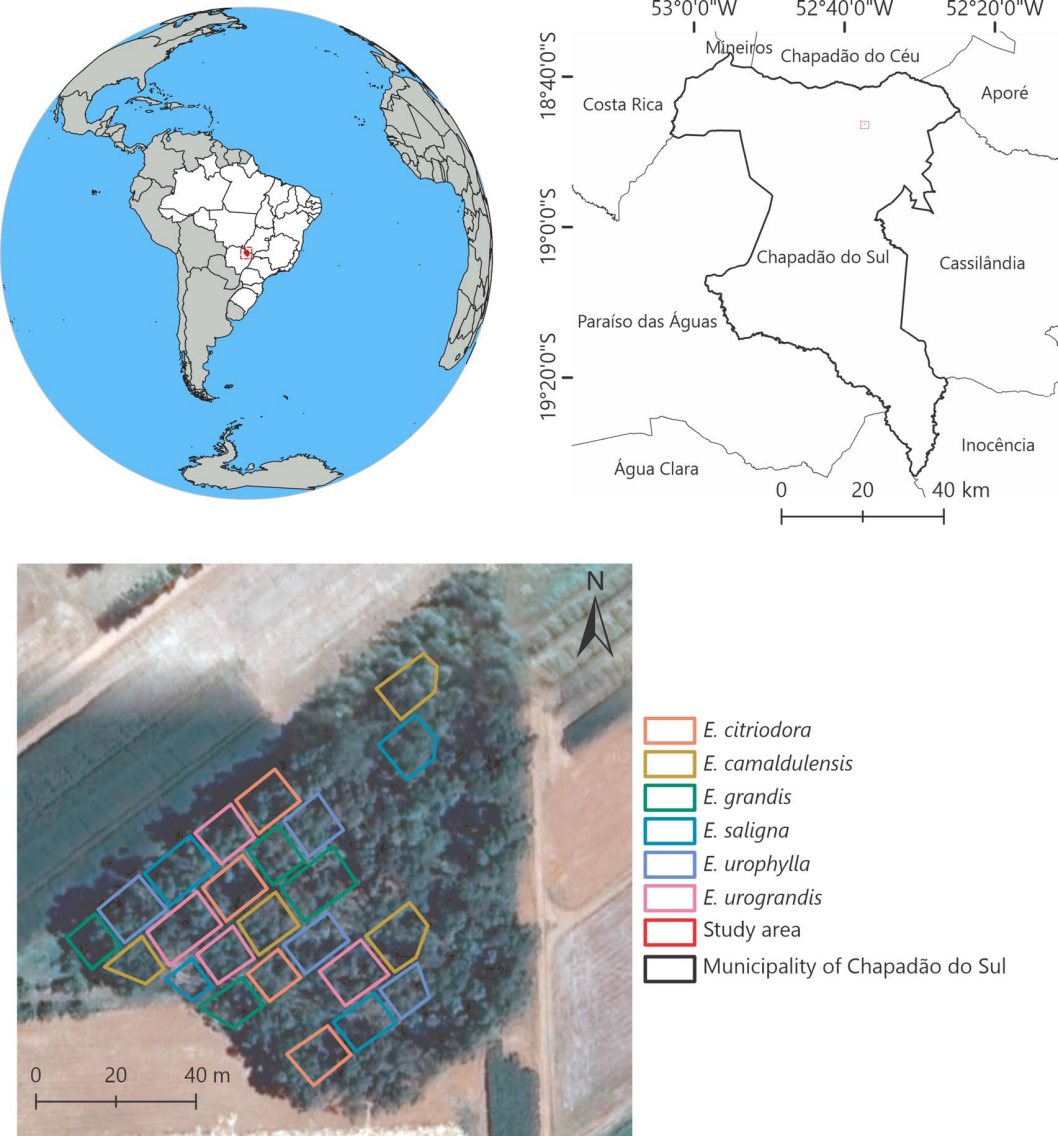
Study area at the Federal University of Mato Grosso do Sul, Chapadão do Sul campus.

This study is conducted in the experimental field of an eucalyptus crop at the Federal University of Mato Grosso do Sul, Chapadão do Sul campus (Fig. 4). According to Köppen, the region is classified as a tropical monsoon climate (Am), with two well-defined seasons, the dry season from April to September and the wet season from October to March^62^. The annual temperature varies from 13 to 28 °C and presents pluviometric precipitation varying from 750 to 1850 mm^63^. The soil in this region is classified as dystrophic red latosol^64^. The plant collection and use following all the relevant guidelines.

### In situ experimental details

Eucalyptus species were monitored in January 2014 in a randomized block experimental design with four repetitions, with 20 plants within each experimental plot. Experimental treatments consisted of six eucalyptus species (*E. camaldulensis*, *E. urophylla*, *E. saligna*, *E. grandis*, *E. urograndis* and *Corymbia citriodora*) and the fertilization needs were determined from the soil chemical analysis, the results of which were obtained: pH (CaCl2): 4.9; organic matter: 31.5 g dm^−3^; phosphor: 13.6 m g dm^−3^; hydrogen + aluminum (H + Al): 5.4; potassium: 0.29 cmolc dm^−3^; calcium: 2.8 cmolc dm^−3^; magnesium: 0.5 cmolc dm^−3^; cation exchange capacity (CEC): 9.0 cmolc dm^−3^; base saturation: 39.9%. The proportions of clay, sand and silt were 46%, 46% and 8%, respectively. Application of herbicides (glyphosate) and pest control was carried out when necessary.

To evaluate the carbon dioxide (CO₂) efflux from the soil (FCO₂), we used the LI-COR portable system, model LI-8100, which monitors the CO₂ concentration variations inside the soil respiration chamber using optical absorption spectroscopy in the infrared spectral region. The soil breathing chamber is a closed system with an internal volume of 854.2 cm^3^ and a circular contact area of 83.7 cm^2^, coupled over the polyvinyl chloride (PVC) collars that were previously inserted into the soil 24 h before collection. The process of placing the PVC collars over the soil surface helps to avoid contamination of the samples by the camera upon contact with the soil, having minimal disturbance of the soil in the measurement process.

Four in situ readings were taken in each block of eucalyptus species, previously defined as equidistant and georeferenced, totaling 96 sample points, where the blocks were divided into quadrants. The readings were dependent on PVC collars for gas flux chamber disposal, where these collars were placed in each quadrant centroid. The FCO₂ acquisition was carried out in the morning, from 8 to 10 a.m. The FCO₂ was evaluated at each point by an adjustment of the CO₂ concentration of the inside chamber air as a function of a parabolic regression over the measured period. The measurement mode for the determination of soil CO₂ emission took 90 s at each of the sample points and the CO₂ concentration inside the chamber was measured every 2.5 s, approximately^65^.

The soil temperature (T_s_) was measured using a Digital Spit-Type Thermometer (DELLT DT-625). It consists of a 20 cm rod that is inserted at least 50% into the soil at 5 cm from the location where the PVC collars were previously installed. Similarly, the Soil Moisture (M_s_) was recorded using a TDR—Time Domain Reflectometry (Hydrosense TM, Campbell Scientific, Australia) device, consisting of a probe with two 12 cm rods that were embedded inside the soil at least 50%, perpendicular to the surface, also at 5 cm from the PVC collars. The soil moisture value is derived from the time it takes for an electric current to travel the distance of 32 mm from one rod to the other. The soil temperature and soil moisture assessments were carried out concomitantly with the assessments of soil CO₂ emission.

### UAV-based multispectral image acquisition and processing

**Fig. 5:**
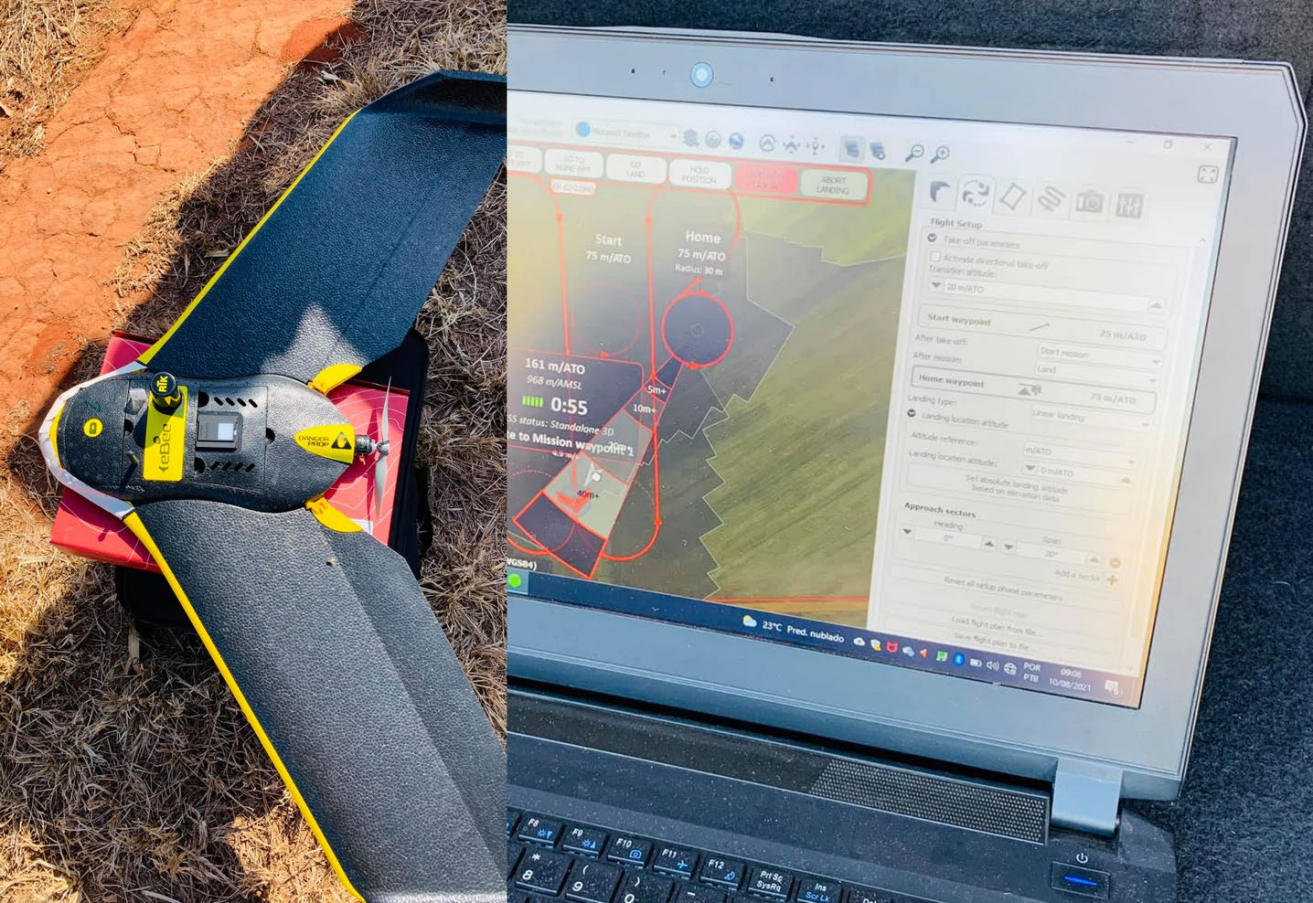
Remotely Piloted Fixed-Wing drone Sensefly eBee RTK loaded with Sensefly Sequoia sensor.

UAV-based multispectral images were obtained simultaneously with the in situ collections using the Parrot Sequoia multispectral sensor coupled to the remotely piloted fixed-wing drone Sensefly eBee RTK, with autonomous flight control (Fig. 5). The flight altitude over the study site was 100 m, providing a spatial resolution of 0.10 m. The Parrot Sequoia is a multispectral camera for agricultural applications, containing a sunlight sensor and an additional 16 Mpx RGB camera for recognition. The sensor has a horizontal field of view (HFOV) of 61.9°, vertical field of view (VFOV) of 48.5° and diagonal field of view (DFOV) of 73.7° and the delivered images are provided in the green (G—550 nm ± 40 nm), red (R—660 nm ± 40 nm), red-edge (RE—735 nm ± 10 nm) and near-infrared (NIR—790 nm ± 40 nm) spectral bands.

Flights were performed with an image lateral and frontal overlap of 80% and 85% respectively, with perpendicular flight lines over the same study area, enhancing the amount and quality of the further processing stages. Increasing the image overlap was necessary to obtain a greater number of scenes containing the same control points. This procedure enabled greater accuracy in the ortho mosaicing of images using Pix4Dmapper software due to the height of the eucalyptus plants and the intense movement of leaves because of the windy conditions. Flights were performed at the zenith in order to minimize shadows on the trees, at 11 a.m. (GMT -4).

The aerial survey was carried out by using RTK (Real Time Kinematics) technology, which in turn allows the estimation of the camera position making it possible to estimate the position of the camera at the instant of image collection with an accuracy of 2.5 cm. The images were mosaicked and orthorectified in the computer program Pix4Dmapper. Field calibration of the Parrot Sequoia was performed in the e-Motion software using images from the reflectance panel for each spectral band right before the UAV takeoff. As the flight duration was less than 15 min, there was no need to repeat the calibration after landing. The positional accuracy of the orthoimages was verified with ground control points (GCP), based on GNSS RTK Emilid Reach.

The processing of the multispectral images was performed using QGIS^66^ software in order to preliminarily analyze the spectral bands and vegetation indices, verifying if the values were correct. Vegetation indices (Table S1) were calculated and overlaid onto the multispectral images and shapefile of the eucalyptus species blocks/quadrant. For each spectral band and vegetation index, the average from all pixels in the blocks/quadrant (Fig. 4) was performed using the extract values by points tool in QGIS. To avoid the border effect between the repetition blocks, an internal buffer of one meter was removed from each block.

### Statistical analysis

First, the data were submitted to descriptive statistics to evaluate the behavior of FCO₂ in the area of eucalyptus plantations and subsequently, an individualized analysis for each species to observe the interrelationships between them. The analyses were performed using R 3.6.3^67^ software.

### Spectral modelling for FCO_2_ estimation

Thus, the CO_2_ flux index model (µmol m^−2^ s^−1^) is the result of the multiplication of NDVI and sPRI, in which there is a relationship between the PRI index, which indicates light-use efficiency in photosynthesis, and NDVI, which indicates the vigor of photosynthetically active vegetation, in which it may be able to capture absorptions from carbon sequestration. Thus, the best correlation is given in Eq. 3^68–70^.

With the purpose of a further examination of the main spectral and emissions of soil carbon dioxide related variables was performed using Pearson correlation (by using the “Corrplot” package)^71^, with a level of significance of *p* < 0.05 at a 95% confidence level. Subsequently, the variables that demonstrated a 95% confidence level with FCO₂ were selected, performing multivariate analysis of variance and canonical variables to investigate the interrelationship between eucalyptus species. For these analyses, the “candisc” package^72^ was used.

### Supplementary Information

Supplementary Information.

## Data Availability

The datasets used and/or analysed during the current study available from the corresponding author on reasonable request.
