# Supplementary material for: Assessing soil CO2 emission on eucalyptus species using UAV-based reflectance and vegetation indices
Source: Sci Rep. 2024 Aug 31;14:20277. doi: 10.1038/s41598-024-71430-2 (PMC11365961; doi:10.1038/s41598-024-71430-2)
Supplement: Supplementary file 1 — Supplementary Information. [file 41598_2024_71430_MOESM1_ESM.docx]

**Supplementary Files**

**
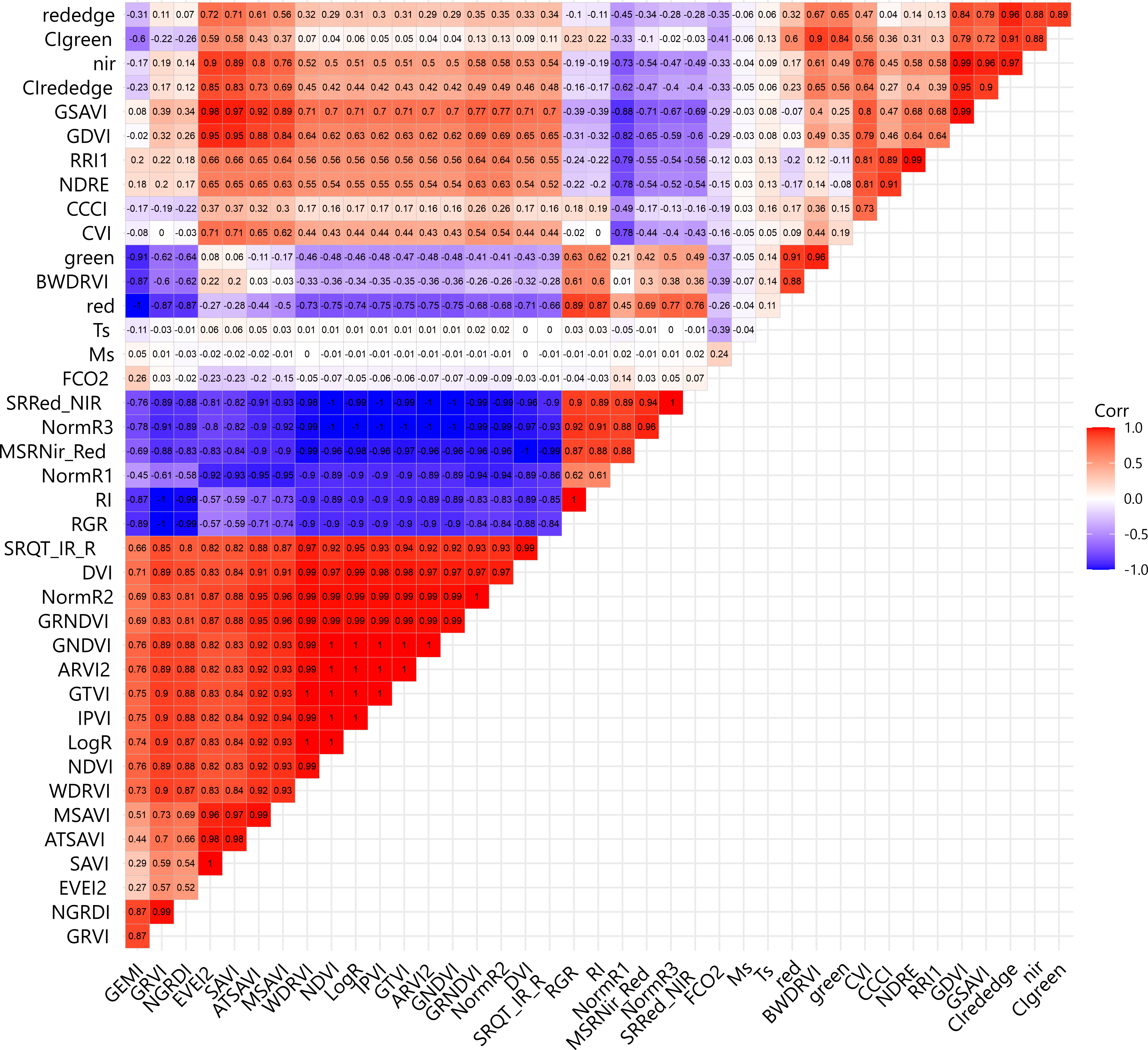
**

**Figure S1.** Pearson’s correlation coefficients compared to vegetation indices, spectral bands and in situ data.

**Table S1.** Calculation of p-values from the correlation for significance level.

|  | **FCO2** | **Ms** | **Ts** | **MSAVI** | **NDRE** | **NDVI** | **SAVI** | **green** | **nir** | **red** | **red-edge** |
| --- | --- | --- | --- | --- | --- | --- | --- | --- | --- | --- | --- |
| **FCO2** | 0 | 0.0169 | 0.0001 | 0.1450 | 0.1490 | 0.5180 | 0.0224 | 0.0002 | 0.0009 | 0.0107 | 0.0004 |
| **Ms** | 0.0169 | 0 | 0.7180 | 0.9610 | 0.7730 | 0.9040 | 0.8550 | 0.6430 | 0.7180 | 0.6690 | 0.5630 |
| **Ts** | 0.0001 | 0.7180 | 0 | 0.7940 | 0.1950 | 0.9170 | 0.5590 | 0.1790 | 0.3670 | 0.2880 | 0.5610 |
| **MSAVI** | 0.1450 | 0.9610 | 0.7940 | 0 | 0 | 0 | 0 | 0.0970 | 0 | 0 | 0 |
| **NDRE** | 0.1490 | 0.7730 | 0.1950 | 0 | 0 | 0 | 0 | 0.4240 | 0 | 0.0898 | 0.1760 |
| **NDVI** | 0.5180 | 0.9040 | 0.9170 | 0 | 0 | 0 | 0 | 0 | 0 | 0 | 0.0038 |
| **SAVI** | 0.0224 | 0.8550 | 0.5590 | 0 | 0 | 0 | 0 | 0.5670 | 0 | 0.0051 | 0 |
| **green** | 0.0002 | 0.6430 | 0.1790 | 0.0970 | 0.4240 | 0 | 0.5670 | 0 | 0 | 0.0000 | 0 |
| **nir** | 0.0009 | 0.7180 | 0.3670 | 0 | 0 | 0 | 0 | 0 | 0 | 0.0938 | 0 |
| **red** | 0.0107 | 0.6690 | 0.2880 | 0 | 0.0898 | 0 | 0.0051 | 0 | 0.0938 | 0 | 0.0015 |
| **red-edge** | 0.0004 | 0.5630 | 0.5610 | 0 | 0.1760 | 0.0038 | 0 | 0 | 0 | 0.0015 | 0 |
